# Detection of Matrix Metalloproteinase Activity by Bioluminescence via Intein-Mediated Biotinylation of Luciferase

**DOI:** 10.3390/s18030875

**Published:** 2018-03-15

**Authors:** Duc Long Nguyen, Hanim Kim, Dasom Kim, Jin Oh Lee, Myung Chan Gye, Young-Pil Kim

**Affiliations:** 1 Department of Life Science, Hanyang University, Seoul 04763, Korea; quyle.dongphuong@gmail.com (D.L.N.); nim93@kaist.ac.kr (H.K.); kds8178@naver.com (D.K.); mayapluszero@nate.com (J.O.L.); 2 Brain Korea 21 Plus BioDefense Research Team, Hanyang University, Seoul 04763, Korea; 3 Research Institute for Natural Sciences, Hanyang University, Seoul 04763, Korea; 4 Research Institute for Convergence of Basic Sciences, Hanyang University, Seoul 04763, Korea

**Keywords:** luciferase, bioluminescence, matrix metalloproteinase, intein, biotinylation

## Abstract

We report bioluminescence analysis of matrix metalloproteinase (MMP) activity in biological substances using a surface-bound luciferase probe. Intein-fused luciferase protein enables site-specific biotinylation of luciferase in the presence of N-terminus cysteine-biotin via intein-mediated splicing process, resulting in a strong association with high bioluminescence signal onto a NeutrAvidin-coated surface. When the peptide substrate for MMP-7 was inserted into a region between luciferase and intein, the biotinylated probe detected MMP-7 activity by cleaving the peptide, and surface-induced bioluminescence signal was strongly reduced in the MMP-secreted media or mouse tissue extracts, compared with that in MMP-deficient control set. Our approach is anticipated to be useful for generating biotinylated proteins and for their applications in diagnosing MMP activity in human diseases.

## 1. Introduction

Cells secrete enzymes into their surrounding extracellular fluids primarily through classical endoplasmic reticulum and Golgi-mediated transportation [1]. Matrix metalloproteinase (MMP) is a protease secreted by cancer and stromal cells and is known to be an important enzyme for cell migration, metastasis and tissue remodeling [2,3]. Quantitative estimation of MMP activity in different tissues or cells provides an important clue to distinguish between normal and abnormal development and also provides a basis for the development of new therapeutic strategies. To date, a zymographic technique has long been used as the standard method for assaying MMP activity [4], which easily detects the activities of different MMPs by degrading the preferential substrate based on the molecular weight. However, it is generally a time-consuming process with protein unfolding and refolding steps and determining the level of MMP activity is difficult, as MMP exists in its complex form with tissue inhibitor of metalloproteinase (TIMP) in the real sample; moreover, sodium dodecyl sulfate (SDS) dissociates TIMPs from MMPs during electrophoresis. To promote rapid and straightforward detection of MMP activity, many methods have been developed based on colorimetry [5], electrochemical measurement [6], or highly sensitive fluorescence measurement using fluorescent dyes [7], proteins [8,9], and nanomaterials [10,11,12]. However, colorimetric or electrochemical signal is critically hindered in real samples and fluorescence signal can also be interrupted by light absorption or scattering in biological samples. To avoid such problems, fluorophores and nanomaterials with long wavelength emission have been employed for in vivo imaging [13,14]; however, complex synthesis, high cost, and low quantum yield are still obstacles for the practical use of fluorescence analysis in real biological samples.

Over the past decades, bioluminescence (BL), generated by luciferase, has been alternatively used for the sensitive and selective detection of targets in most cell and tissue types due to its wide emission range and no need for incident excitation [15,16]. While BL-based detection has been mostly focused on intracellular sensing, such as bacterial sensor, gene reporter, or bioluminescence resonance energy transfer (BRET) system for protein-protein interactions, luciferase and its mutant with prolonged stability have been implemented for in vitro analysis of protease activity in solutions [17,18,19]. Despite the versatile application of luciferase, the development of affinity-based method on a surface is still needed to extend BL measurement into throughput analysis in real sample. Because the avidin-biotin conjugate is stronger than metal or antibody affinity, it is possible to easily capture the luciferase probe on the chip surface in a complex media, given the effective strategy to introduce low molecular biotin into a single luciferase protein.

Here, we report a surface-captured luciferase probe via biotin-streptavidin interaction for the detection of MMP activity in cell culture media and tissue extracts. C-terminus-specific biotinylation of luciferase protein was achieved by intein-mediated splicing method. Intein protein is observed in all domains of life as an internal protein element, which is required for ligating two flanking peptide sequences, and has been implemented in protein purification, protein semi-synthesis, and protein modification in the field of biotechnology [20,21,22,23,24]. For C-terminus biotinylation of the luciferase protein, the recombinant luciferase protein with peptide substrate and intein (luciferase-peptide-intein) was reacted with cysteine-lysine-biotin (CK-biotin) in the presence of a nucleophile thiol reagent (2-mercaptoethane-sulfonic acid), to yield a biotinylated luciferase probe (luciferase-peptide-biotin) via intein-mediated ligation. We monitored MMP-7 activity by capturing the biotinylated probe from real samples, including MMPs, using a NeutrAvidin (NA)-coated microplate. In addition, we tested the culture media of cancer cells and mouse tissue extracts to compare the different levels of MMP-7 activity. BL intensity of target sample on a microplate was normalized to that of the MMP-free sample.

## 2. Materials and Methods

### 2.1. Materials

Active matrix metalloproteinase-7 (MMP-7) and matrix metalloproteinase-2 (MMP-2) were purchased from Merck Millipore (Darmstadt, Germany). Coelenterazine-*h* was from Nanolight Technologies (Pinetop, AZ, USA). Ni(II)-nitrilotriacetic acid (Ni-NTA) resin and NA-coated white 96-well plate were from Thermo Fisher Scientific Inc. (Waltham, MA, USA). L-arabinose, 2-mercaptoethane-sulfonic acid (MESA), and L-Cysteine (L-Cys) were from Sigma-Aldrich (Yongin, Korea). Cys-Lys-Biotin (CK-Biotin) was synthesized by Peptron Inc. (Daejeon, Korea). RIPA buffer was purchased from Cell Signaling Technology (Danvers, MA, USA).

### 2.2. RLuc8-pep-GyrA Plasmid Construction

To insert MMP-7 peptide substrates (GGVPLSLTMGG termed as m7) between *R*Luc8 (encoding luciferase mutant 8 from *Renilla reniformis*) and GyrA (encoding Mxe GyrA intein from *Mycobacterium xenopi*), m7pep-GyrA gene was constructed using the pBAD-*R*Luc8-GyrA plasmid [17,25] by consecutive PCR using four forward primers (1st 5′-ACA ATG GGT GGT GAA TTC TGC ATC ACG-3′, 2nd 5′-TA CCT CTG TCA CTG ACA ATG GGT GGT G-3′, 3rd 5′-C GAG GGA GGA GTA CCT CTG TCA CT-3′, and 4th 5′-CCG CTC GAG GGA GGA GTA CCT-3′) and single common reverse primer (5′-ATGC GAA TTC ACC ACC GCT ACG CAG ACT TAC AAT ACC ACC CTG CTC GTT CTT CAG-3′). The PCR products were digested with combinations of Xho I/Hind III and ligated into the same enzyme-digested pBAD-*R*Luc8-GyrA to yield pBAD-*R*Luc8-m7-GyrA plasmids.

### 2.3. Preparation for RLuc8-m7-Bio and Rluc8-pep-C via Intein-Mediated Ligation

To obtain the fusion protein with a His_6_-tag at its C-terminus, the plasmid was transformed into *E*. *coli* TOP10 competent cells and the transformed cells were cultured for 16 h at 37 °C in 0.5 L of Luria–Bertani broth containing 100 µg mL^−1^ ampicillin with reciprocal shaking (200 rpm min^−1^) until the optical density of the solution reached 0.7. To induce protein expression, 0.2% arabinose was added to the cultures, and the cultures were further incubated at 37 °C for 5 h. The cells were harvested by centrifugation and suspended in 30 mL of lysis buffer (50 mM phosphate-buffered saline (PBS) containing 10 mM imidazole, 300 mM NaCl, and 1 mg mL^−1^ lysozyme; pH 8.0), followed by sonication for 10 min with a 10 s on–off cycle. After centrifugation (5200× *g*) for 30 min at 4 °C, the supernatants were collected and filtered using a syringe filter (Minisart) with a 0.45 µm pore size. The soluble lysate was then mixed with 1 mL of 50% Ni-NTA agarose slurry (Thermo Scientific) equilibrated in lysis buffer. The mixture was incubated for 16 h at 4 °C with reciprocal shaking to promote efficient binding. For purification, the lysate-Ni-NTA mixture was loaded into a 5-mL column (Thermo Scientific) with the bottom outlet capped, and the flow-through sample was collected just after removing the bottom outlet. The column was rinsed four times with the washing buffer (50 mM PBS containing 300 mM NaCl, and 20 mM imidazole; pH 8.0) and the protein was eluted from the column using an elution buffer (50 mM PBS containing 300 mM NaCl and 500 mM imidazole; pH 8.0). The eluted fractions were further purified using the PD-10 desalting column (GE Healthcare), and the purified protein was concentrated and resuspended in 100 mM PBS (pH 7.4) using Microcon (YM-50, Millipore). Protein concentration was determined by measuring absorbance at 280 nm and using an extinction coefficient of 91,010 M^−1^ cm^−1^. To generate *R*luc8-m7-Bio (*R*luc8-GGVPLSLTMGGC-Biotin) or its unbiotinylated form (*R*luc8-GGVPLSLTMGGC), the splicing process of intein-fused protein (*R*luc8-m7-GyrA-His_6_) was performed using an aminothiol derivative. Briefly, for the construction of *R*luc8-m7-Bio, MESA (40 μL at 50 mM) and CK-Biotin (20 μL at 1 mM) were mixed with the purified *R*luc8-m7-GyrA-His_6_ protein (40 μL at 50 µM) in the reaction buffer (0.1 mM PBS, pH 8.0). For the construction of *R*luc8-m7-C, L-Cys (20 μL at 1 mM) was used instead of CK-biotin. The reaction mixture was incubated overnight at 4 °C with gentle shaking in the dark. To purify the cleaved product, 200 μL of the reactant was mixed with a 50% Ni-NTA agarose slurry (100 μL) for 1 h at 4 °C with reciprocal shaking to promote efficient binding. After centrifugation of the agarose mixture, the supernatant protein was further purified using Microcon (YM-30, Millipore) to remove excess MESA, CK-biotin, or L-Cys. The desired product (*R*Luc8-m7-Bio or *R*Luc8-m7-C) was confirmed using SDS-PAGE and quantified by measuring absorbance at 280 nm using an extinction coefficient of 63,495 M^−1^ cm^−1^.

### 2.4. Sample Preparation

HT-1080 (human fibrosarcoma, MMP-7-positive) and HT-29 (human colorectal adenocarcinoma, MMP-7-negative) were separately cultured onto a T75 tissue culture flask (SPL Life Science, Korea) at 37 °C in 10% fetal bovine serum (FBS)-containing culture medium (RPMI 1640). After two days, the cell culture was refreshed with serum-free medium, which was followed by further incubation for 24 h. The culture media were then transferred to a 15-mL Falcon tube using a sterile pipette. The supernatant was obtained and concentrated by centrifugation (6800× *g*, 10 min) using Centricon (Amicon Ultra-4 Centifugal Filters 10 K, Merck Millipore, Darmstadt, Germany). Mouse tissue extracts were obtained by sonicating the tissue samples for 5 s at 4 °C in PBS containing 1% Triton-X-100 without protease inhibitors. The soluble fraction was stored at −80 °C in a freezer for long-term storage. Total protein concentration in the cultured medium or tissue extract was determined using the Bradford method according to the manufacturer’s instructions (Bio-Rad, Hercules, CA, USA).

### 2.5. Determination of Biotinylation and Unbiotinylation of Proteins Using Biolayer Interferometry

To determine the biotinylation of purified proteins, surface analysis was performed by bio-layer interferometry (BLItz instrument, ForteBio Inc., Menlo Park, CA, USA). Prior to binding measurements, a streptavidin-coated optical probe (#18-5019, Fortebio, USA) was equilibrated for 10 min in the loading buffer (PBS containing 0.2 mg mL^−1^ bovine serum albumin (BSA)). After obtaining the initial base line for 60 s, *R*luc8-m7-Bio (4 µL at 1 µM in PBS) was loaded onto the streptavidin-coated sensor for 180 s. The biosensor was washed for 120 s by flowing through PBS. As a negative control, *R*luc8-m7-C (4 µL at 1 µM in PBS) was then associated for 180 s, followed by dissociation for another 120 s in PBS.

### 2.6. MMP Assays on the Microplate

Each well in the NA-coated plate was pretreated with TBS buffer containing 0.1% BSA, 0.05% Tween-20 (pH 7.4) for surface passivation and activation. *R*Luc8-m7-Bio (90 μL at 70 µg mL^−1^, which is equivalent to 1.94 µM) in the reaction buffer (20 mM Tris containing 5 mM CaCl_2_, and 100 mM NaCl; pH 7.6) was mixed with MMP enzymes (10 μL at each concentration in the reaction buffer). For measuring the MMP activity in real samples, *R*Luc8-m7-Bio (80 μL at 70 µg mL^−1^) in the reaction buffer was mixed with 20 μL of the culture media or tissue lysate (50 µg mL^−1^ of total protein concentration). As a control for measuring the real sample, the probe was incubated with 20 μL of BSA (50 µg mL^−1^) under the same condition. One hundred microliters of the mixed solution were then incubated for 2 h at 37 °C. The reactant (95 μL) was added to each well in the NA-coated plate for 1 h at room temperature to induce biotin-streptavidin association. The surface was washed three times with the washing buffer. To obtain BL, 100 μL of PBS was initially added to each well, followed by addition of coelenterazine-*h* (100 μL at 2 μg mL^−1^ in PBS). BL spectrum was acquired from 400–650 nm using a multimode plate reader (Varioskan, Thermo Scientific, Inc., USA). BL image was obtained using a chemiluminescence/BL image acquisition system (Fusion SL, Vilber Lourmat, Torcy, France).

## 3. Results and Discussion

### 3.1. Intein-Mediated Site-Specific Biotinylation of Luciferase

**Scheme 1:**
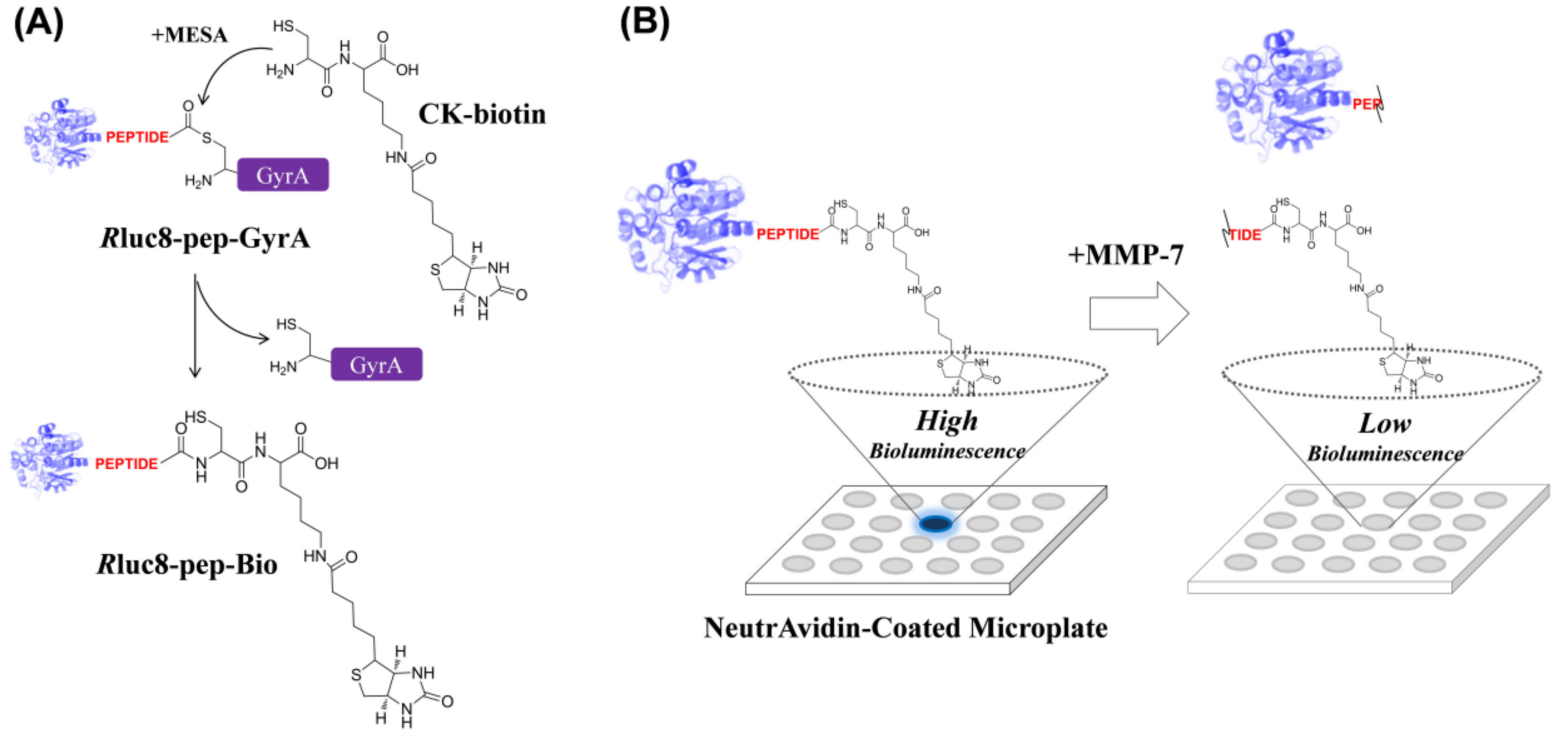
Schematic representation of bioluminescence (BL)-based assay of matrix metalloproteinase (MMP) activity on a microplate. (**A**) Intein-mediated biotinylation of *Renilla* luciferase mutant protein (*R*luc8). Recombinant luciferase protein with peptide substrate and GyrA intein (*R*luc8-pep-GyrA) was biotinylated at the C-terminus via a self-splicing process between intein and CK-biotin. (**B**) BL-based assay of MMP-7 activity via NA-biotin interaction on the microplate surface. As the concentration of active MMP-7 increases, the BL signal decreases, due to the loss in luciferase activity. For BL generation, coelenterazine-*h* was used as the substrate for luciferase.

For site-specific introduction of a biotin moiety into luciferase, we performed an intein-mediated ligation method using a *Renilla* luciferase mutant (termed as *R*luc8), which exhibits higher stability in serum than in wild-type luciferase [26]. An intein protein found in *Mycobacterium xenopi* GyrA gene (*Mex* GyrA) [27] undergoes a self-splicing process to produce stable α-thioester in the presence of CK-biotin and MESA, leading to the ligation of CK-biotin into the C-terminus of *R*luc8 when intein is fused at the C-terminus of *R*luc8. MESA served as a nucleophilic compound to facilitate intein-mediated protein splicing [20]. As illustrated in Scheme 1A, recombinant *R*luc8, containing the peptide substrate for MMP, was expressed in *E. coli* (*R*luc8-pep-GyrA) to obtain the biotinylated form via intein-mediated ligation process (*R*luc8-pep-Bio). As shown in Scheme 1B, the purified *R*luc8-pep-Bio protein can be devised to monitor MMP activity, wherein the probe can be strongly captured on a NA-coated microplate surface after MMP reaction with the real sample. The BL signal was generated on the surface in the presence of the luciferase substrate (coelenterazine-*h*), and the signal was inversely proportional to the amount of MMPs in samples. This site-specific biotinylation of luciferase via intein-mediated ligation is very efficient for detecting protease activity because this process results in a single conjugation at a 1:1 ratio of luciferase-peptide to biotin; that is, the biotin group can be easily removed from the protein by a small amount of protease activity. In contrast, in the case that luciferase is randomly conjugated with peptide-biotin via classical covalent reaction, the multiple biotin groups on the luciferase will not be fully removed even after protease activity, leading to no signal difference on a NA-coated surface.

**Figure 1:**
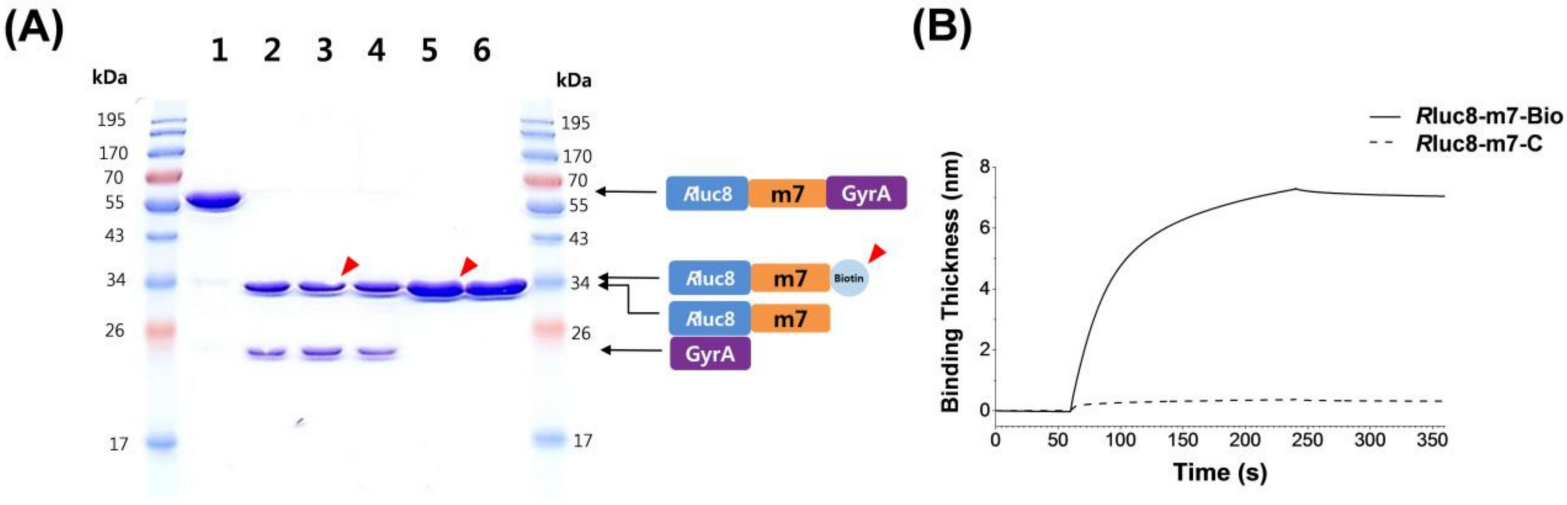
(**A**) SDS-PAGE analysis of intein splicing of *R*luc8-m7-GyrA: control protein (*R*luc8-m7-GyrA, lane1), protein + MESA (lane 2), protein + MESA + CK-biotin (lane3), protein + MESA + L-cysteine (lane 4), purified protein of lane 3 (lane 5), and purified protein of lane 4 (lane 6). (**B**) Bio-layer interferometric analysis of *R*luc8-m7-Bio and *R*luc8-m7-C (control). The probe (4 µL at 0.5 µM) was flooded for 6 min on a streptavidin-coated optical sensor.

To verify intein-mediated biotinylation of *R*luc8, we performed sodium dodecyl sulfate polyacrylamide gel electrophoresis (SDS-PAGE) and bio-layer interferometry (Figure 1). When the recombinant protein (*R*luc8-m7-GyrA), containing the peptide substrate (GGVPLSLTMGG) for MMP-7 [28], was treated with MESA alone (lane 2) or with MESA with cysteine-labeled ligands (CK-biotin (lane 3) and L-cysteine (lane 4); Figure 1A), the reactant showed two splicing protein fragments in SDS-PAGE (lanes 2–4), compared with the original protein (lane 1). To compare the attachment of biotin via the trans-thioesterification process between the intein and cysteine derivative, L-cysteine (C) without a biotin moiety was used as the negative control under the same intein-splicing condition. After the purification of products using Ni-NTA agarose beads to remove His_6_-tagged proteins in the reaction mixture (His_6_-tag was ligated to the C-terminus of GyrA intein protein), no difference was observed in gel mobility between putative *R*luc8-m7-Bio (lane 5) and *R*luc8-m7-C (lane 6), which was probably due to a minor difference in the molecular weight of the two products. When the biotinylated and unbiotinylated protein were flooded onto the streptavidin-coated surface for bio-layer interferometry, it was observed that the binding thickness increased with the saturated curve after 4 min, indicating the strong association of *R*luc8-m7-Bio onto the surface (solid line in Figure 1B). In contrast, no significant binding was observed for *R*luc8-m7-C (dashed line in Figure 1B). This result strongly indicated that the biotin group was successfully introduced to the C-terminus of *R*luc8-m7-GyrA via intein-mediated splicing process to yield *R*luc8-m7-Bio.

### 3.2. Assaying MMP-7 Activity

**Figure 2:**
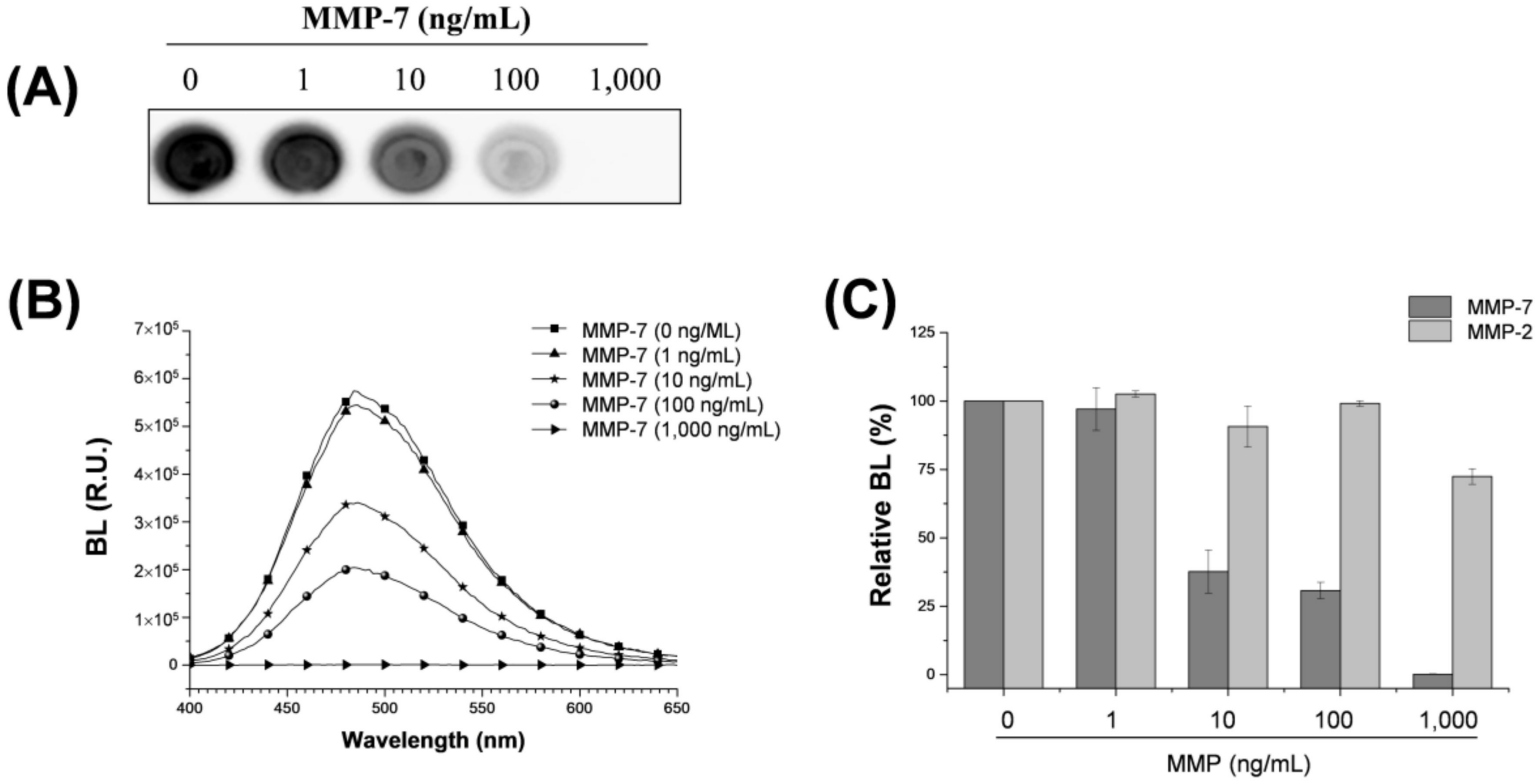
Assay of MMP-7 activity by BL using the *R*luc8-m7-Bio probe onto the NA-coated surface. BL images (**A**); and BL spectra (**B**) of the probe were obtained as a function of active MMP-7 concentration (0–1000 ng/mL). (**C**) Bar graph represents relative BL intensity in response to MMP-7 (dark gray) and MMP-2 (light gray) enzymes over the same range of MMP concentration. Standard deviation was obtained from four independent experiments.

We investigated whether the biotinylated luciferase probe can detect MMP-7 activity. MMP-7, also known as matrilysin, was chosen as the model protease because it exhibits broad specificity for diverse substrates in the extracellular matrix, including casein, gelatins, fibronectin, and proteoglycan [29], and it has also been implicated in promoting tumor invasion associated with MMP-2 and MMP-9 [30]. *R*luc8-m7-Bio was incubated in 10-fold increments over various concentration ranges of active MMP-7 (0–1000 ng mL^−1^), which was followed by affinity-based immobilization onto the NA-coated microplate. As shown in Figure 2, the signal intensity of BL image decreased notably with the increase in the concentration of active MMP-7 (Figure 2A), which was in accordance with the signal change in the BL spectra of *R*luc8-m7-Bio as a function of MMP-7 concentration (Figure 2B). In contrast, MMP-2 showed no significant signal reduction within the same concentration range (Figure 2C). Although the peptide substrate for MMP-7 exhibited high specificity for MMP-7, rather than for MMP-2, a slight signal reduction was observed at high concentrations of MMP-2, indicating this peptide substrate has low cross-reactivity to MMP-2. Detection limit of MMP-7 activity was determined between 1 and 10 ng mL^−1^ of MMP-7, which showed similar [12] or slightly improved [5,31] results when compared with other previously reported assays. The peptide substrate (VPLSLTMG) used in this study is known to have a 10-fold higher *k*cat/*K*_M_ value in MMP-7 than that in MMP-2 or MMP-9 [28]. This result indicates that *R*luc8-m7-Bio is useful to quantitatively detect MMP-7 activity with high specificity and sensitivity.

**Figure 3:**
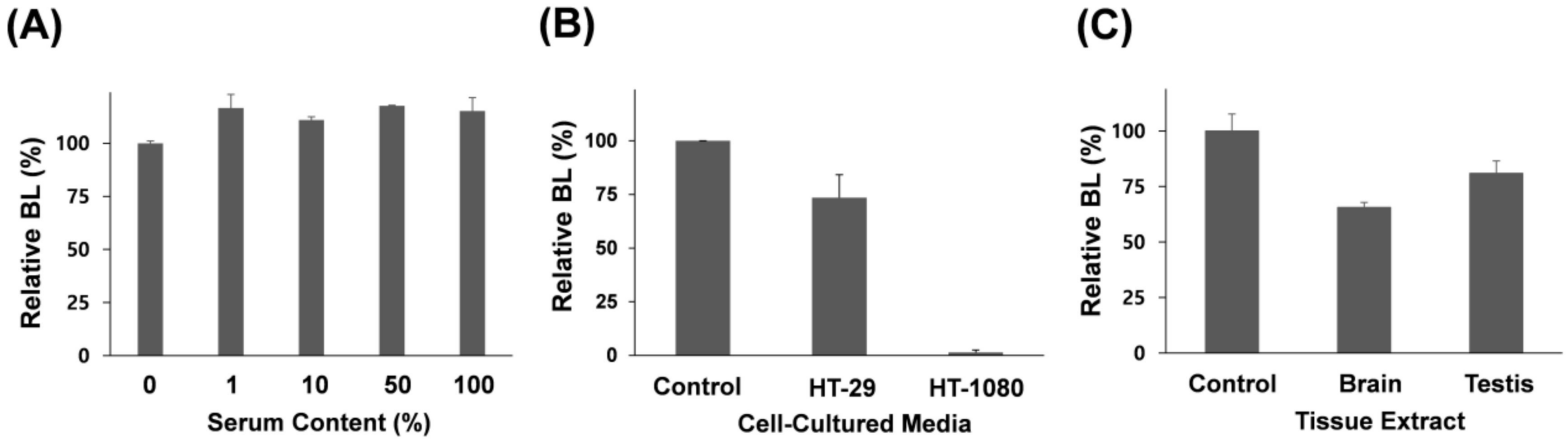
Assay of MMP-7 activity by BL in biological samples using the *R*luc8-m7-Bio probe onto the NA-coated surface. (**A**) Changes in BL intensity as the serum content in cultured media increases. Relative BL signals: in the cultured media of cancer cells (HT-29 and HT-1080) (**B**); and in mouse tissue extracts (brain and testis) (**C**). Bar graphs represent relative BL intensity in response to MMP-7 activity at the same total protein concentration (50 µg mL^−1^). Standard deviation was obtained from three independent experiments.

To examine whether the luciferase functions accurately in real biological samples, we attempted to determine whether the BL signal remains unchanged at various concentrations of mouse serum. After mouse serum without active MMPs was mixed with *R*luc8-m7-Bio for 2 h, the probe was captured onto the NA-coated surface and washed three times with buffer. As a result, there was no significant reduction in BL at various concentrations of the mouse serum (Figure 3A), although a slight change in BL was observed, indicating that the luciferase probe is stable against serum and BL-based measurement on a surface is useful for monitoring MMP activity in complex substances. To further examine whether this method can detect MMP-7 activity in biological substances, we tested this probe in cultured media (total protein concentration was 50 µg mL^−1^) of two cancer cells (Figure 3B): HT-1080 (a human fibrosarcoma cell line that secretes MMP-7) and HT-29 (a human colorectal adenocarcinoma cell line that does not secrete MMP-7). To measure the relative MMP activity, the same BSA concentration (50 µg mL^−1^) used for cell culture was used as the negative control. When the cell culture media containing the same number of cells was reacted with our probe and analyzed on the microplate, a strongly reduced signal (high MMP-7 activity) was observed in the cultured medium of HT-1080 when compared with that in the control set. In contrast, a slightly reduced signal (low MMP-7 activity) was observed in the cultured medium of HT-29. In addition, when mouse tissue extracts (total protein concentration was 50 µg mL^−1^), including brain and testis, were tested using the same protocol, a relatively low activity level of MMP-7 was observed (Figure 3C), compared to that in HT-1080-cultured media, which is in agreement with a previous report [32]. It is important to note that membrane-type MMPs (MT-MMPs), rather than secreted MMP-7, in the brain and testis are predominant [33], wherein latent MT-MMPs are exclusively activated by environmental factors, such as other proteases, or growth factors to accommodate rapid remodeling events of the extracellular matrix in the brain and during embryonic development. Because these biological samples were collected from normal mice, and MMP-7 is correlated with tissue injury and viral infection, further studies on a disease mouse model are required to elucidate the functional role of MMP-7 activity in tissue extracts. Most importantly, these results indicate that our method is very effective to detect MMP-7 activity in biological substances.

Taken together, our BL-based method has many advantages over other colorimetric or fluorescence methods. The highly stable luciferase probe enables detection of specific enzyme activity in complex samples, such as sera and tissue extracts, without autofluorescence or other background noises. Biotinylated luciferase via intein-mediated splicing process provides a surface-immobilized strategy to rapidly read BL intensity. Importantly, unlike classical zymography based on gel electrophoresis, which is limited to a couple of MMPs and is a time-consuming process, this method based on a peptide substrate has great potential for a wide range of proteases by a simple process. Although this assay is a signal-off measurement method in which the BL signal decreases with increasing MMP activity, it is possible to use a control to accurately compare the relatively reduced signal and to use the difference as the positive correlation. In combination with long life-time luciferase and automation technique of sample reaction and washing step in the following study, this method is expected to be useful for monitoring and comparing various protease activities in biological samples in a high-throughput and multiplex manner.

## 4. Conclusions

In this study, we demonstrate a BL-based assay of MMP activity in real biological samples using surface-bound luciferase. A biotinylated luciferase probe (*R*luc8-m7-Bio), which was generated via intein-mediated splicing process, was used to monitor BL in response to MMP-7 activity on NA-coated surface. Upon mixing the probe with biological samples containing active MMP-7, the resulting surface-bound luciferase probe enabled to discriminate MMP-7 activity in cell-cultured media or mouse tissue extracts with high stability of luciferase. Owing to its applicability in biological substances, we anticipate that our approach can be applied for monitoring protease activity in biological substances in a simple manner.
